# p20BAP31 Induces Autophagy in Colorectal Cancer Cells by Promoting PERK-Mediated ER Stress

**DOI:** 10.3390/ijms25105101

**Published:** 2024-05-07

**Authors:** Xiaohan Jiang, Guoxun Li, Benzhi Zhu, Jiaying Yang, Shuyu Cui, Rui Jiang, Bing Wang

**Affiliations:** College of Life and Health Science, Northeastern University, 195 Chuangxin Road, Hunnan District, Shenyang 110819, China; xiaohanjiang2014@163.com (X.J.); liguoxuner@126.com (G.L.); zhubenzhi0203@163.com (B.Z.); 2201476@stu.neu.edu.cn (J.Y.); cuishuyu0615@163.com (S.C.)

**Keywords:** p20BAP31, autophagy, PI3K/AKT/mTOR, ER stress, apoptosis, colorectal cancer

## Abstract

B-cell receptor-associated protein 31 (BAP31) is an endoplasmic reticulum (ER) membrane protein involved in apoptosis and autophagy by communication with ER and mitochondria. BAP31 is cleaved by caspase-8 and generates a proapoptotic fragment, p20BAP31, which has shown to induce ER stress and apoptosis through multiple pathways. In this study, we found that p20BAP31 significantly increased the agglomeration of LC3 puncta, suggesting the occurrence of autophagy. Therefore, it is meaningful to explore the mechanism of p20BAP31-induced autophagy, and further analyze the relationships among p20BAP31-induced autophagy, ER stress and apoptosis. The data showed that p20BAP31 induced autophagy by inhibition of the PI3K/AKT/mTOR signaling in colorectal cells. ER stress inhibitor 4-PBA and PERK siRNA alleviated p20BAP31-induced autophagy; in turn, autophagy inhibitors 3-MA and CQ did not affect p20BAP31-induced ER stress, suggesting that p20BAP31-induced ER stress is the upstream of autophagy. We also discovered that ROS inhibitor NAC inhibited p20BAP31-induced autophagy. Furthermore, inhibition of autophagy by CQ suppressed p20BAP31-induced apoptosis and ameliorated cell proliferation. Importantly, p20BAP31 markedly reduced the tumor size in vivo, and significantly enhanced the autophagy levels in the tumor tissues. Collectively, p20BAP31 initiates autophagy by inhibiting the PI3K/AKT/mTOR signaling and activating the PERK-mediated ROS accumulation, further promotes p20BAP31-induced apoptosis and ultimately results in cell death. This study comprehensively reveals the potential mechanism of p20BAP31-induced cell death, which may provide new strategies for antitumor therapy.

## 1. Introduction

B-cell receptor-associated protein 31 (BAP31) is an integral endoplasmic reticulum (ER) membrane protein [1,2], which has been reported to transport multiple transmembrane proteins [3,4,5,6] and is involved in apoptosis [7,8,9] and autophagy [10,11]. After activation of cell surface death receptors, BAP31 is cleaved by caspase-8, generating a proapoptotic fragment, p20BAP31. p20BAP31 has shown to stimulate the release of ER Ca^2+^ and cytochrome c (cyt.c) [12], and initiate a paraptosis-like cell death pathway [13]. Our recent study demonstrated that p20BAP31 induces cell apoptosis via both the AIF caspase-independent and the ROS/JNK mitochondrial pathways [14]. In conclusion, the ability of p20BAP31 to induce cell death through multiple pathways makes it potentially valuable in reducing drug resistance in tumor cells. BAP31 plays a vital role in cell death mainly due to the cleavage of BAP31, this process can be divided into two aspects, BAP31 deletion and the generation of p20BAP31. Although previous studies have shown that the loss of BAP31 induces autophagy [11], whether p20BAP31 induces autophagy and the mechanism of p20BAP31-induced autophagy needs to be further investigated.

Autophagy is an evolutionarily conserved physiological process, playing vital roles in development, differentiation, immune defense and the suppression of tumorigenesis [15,16,17]. Autophagy plays an essential pro-survival mechanism in cellular adaptation to starvation or stress conditions [18,19]; enforced overactivation of autophagy caused cell death in certain contexts [20]. The autophagy process is divided into three stages: induction, vesicle nucleation and elongation [21]. Upon activation by ATG7 during autophagy, cytoplasmic soluble LC3B-I undergoes ubiquitin-like modification, ATG12 conjugates ATG5 and promotes the conjugation of LC3B-I to phosphatidylethanolamine (PE) on the autophagic membrane, forming LC3B-II [22]. LC3B-II binds to the membrane of phagocytes, and is frequently regarded as a unique marker of autophagosomes to measure autophagic activity [23,24]. Moreover, one of the central checkpoints of negatively regulated autophagy is mTOR, and inhibition of PI3K-AKT-mTOR signaling significantly stimulate autophagy and restrain tumor growth [25,26,27]. Therefore, to identify whether p20BAP31 induces autophagy and the mechanism of p20BAP31-induced autophagy, we detected the effects of p20BAP31 on autophagy marker protein and PI3K-AKT-mTOR signaling.

Programmed cell death (PCD) is crucial for animal development, tissue homeostasis and pathogenesis. There are several PCD mechanisms, namely apoptosis, autophagy and necroptosis, etc. [28] Moreover, the functional relationship between autophagy (‘self-eating’) and apoptosis (‘self-killing’) is complex; autophagy and apoptosis may be triggered by common upstream signals, and coexist to induce cancer cell death [29,30]. In other cases, the cell alternates between the two responses in a mutually exclusive manner [31,32]. Therefore, it is necessary to explore the relationship between p20BAP31-induced apoptosis and autophagy. In addition, numerous studies have suggested that ER stress is a common cellular stress response that is closely related to the activation of autophagy, which is an important evolutionarily conserved mechanism for maintaining cellular homeostasis [21,33,34]. To alleviate ER stress and restore ER function, cells have an integrated signaling system that includes the unfolded protein response (UPR) and endoplasmic reticulum-associated degradation (ERAD) [35]. The role and molecular mechanism of ER stress-mediated autophagy were mainly discussed in different conditions, such as hypoxia, ischemia/reperfusion, tumor and so on [36,37]. Considering that previous study has demonstrated that p20BAP31 causes ER stress, we intend to further explore the relationship between p20BAP31-induced ER stress and autophagy.

This study aimed to investigate the mechanism of p20BAP31-induced autophagy, and the relationships among p20BAP31-induced autophagy, ER stress and apoptosis. We found that p20BAP31 induced autophagy of colorectal cells in vitro and in vivo. p20BAP31-induced autophagy is mediated by downregulation of the PI3K/AKT/mTOR pathway. Furthermore, p20BAP31-induced apoptosis was inhibited by autophagy inhibitor chloroquine (CQ), which implies that p20BAP31-induced autophagy promotes apoptosis. Interestingly, p20BAP31-induced autophagy can be inhibited by ER stress inhibitor 4-PBA or siPERK; however, p20BAP31-induced ER stress cannot be suppressed by autophagy inhibitors 3-methyladenine (3-MA) or CQ, which means that p20BAP31-induced ER stress maybe the upstream of autophagy. Collectively, these findings revealed that p20BAP31-induced ER stress is crucial for p20BAP31-induced autophagy, and p20BAP31-induced autophagy promotes p20BAP31-induced apoptosis, which eventually leads to cell death. This study comprehensively revealed the potential mechanism of p20BAP31 induced cell death, which may provide new strategies for antitumor therapy.

## 2. Results

### 2.1. Overexpression of p20BAP31 Induces Autophagy

**Figure 1:**
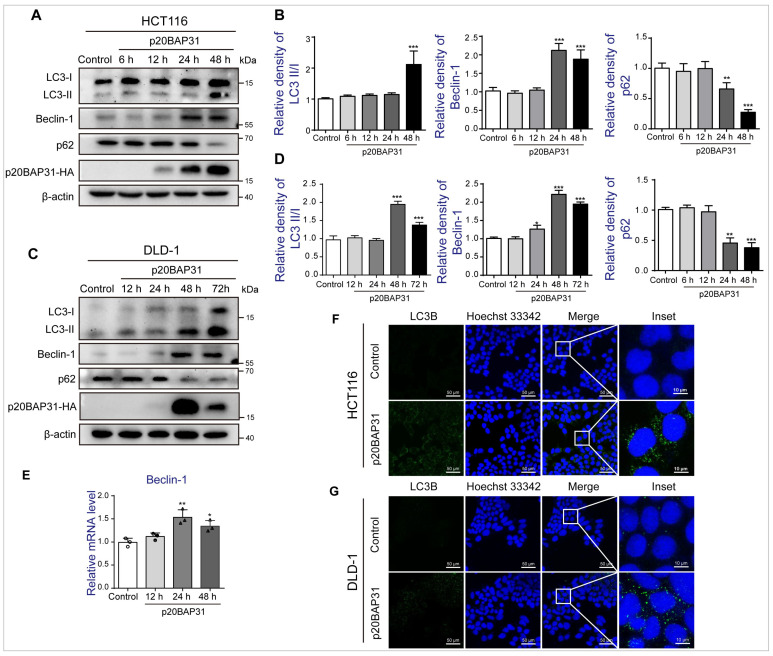
Overexpression of p20BAP31 induces autophagy. (**A**,**B**) HCT116 cells were transfected with p20BAP31 for the indicated time periods. The levels of LC3, Beclin-1, p62 and p20BAP31-HA were analyzed by Western blot. β-actin was used as a loading control. (**C**,**D**) DLD-1 cells were transfected with p20BAP31 for the indicated time periods. The levels of LC3, Beclin-1, p62 and p20BAP31-HA were analyzed by Western blot. β-actin was used as a loading control. (**E**) The mRNA level of Beclin-1 was detected by real-time PCR after transfected with p20BAP31 for the indicated time periods in HCT116 cells. GAPDH served as a loading control. (**F**,**G**) HCT116 and DLD-1 cells were transfected with p20BAP31 for 48 h. Immunofluorescence staining of the autophagic marker LC3B (green). Hoechst 33342 (blue) was used to visualize cell nuclei. Representative images were captured using confocal immunofluorescence microscopy. The data are presented as means ± SDs, *n* = 3 replicates, by one-way ANOVA analysis. * *p* < 0.05, ** *p* < 0.01, *** *p* < 0.001.

To explore whether p20BAP31 induces autophagy, we first compared the morphology of autophagosomes induced by p20BAP31, and found that GFP-mCherry-LC3 puncta appeared to be obviously agglomerated in p20BAP31-overexpressed HCT116 cells (Figure S1A,B). Massive red puncta in p20BAP31-overexpressed HCT116 cells clearly showed an accumulation of autolysosomes, suggesting impairment in auto-lysosomal degradation of autophagic cargo. During autophagy, LC3B-I is converted to LC3B-II through the insertion of phosphatidylethanolamine (PE), and then recruited into the forming phagophore membrane [38]. Therefore, we detected the expression of autophagy marker proteins LC3, Beclin-1 and the autophagy substrate protein p62 at different time points. The ratio of LC3II/LC3I was significantly increased after transfected with p20BAP31 for 48 h both in HCT116 and DLD-1 cells. p20BAP31 also enhanced the expression level of Beclin-1 both in HCT116 and DLD-1 cells, a protein necessary for the initiation of autophagosome formation during autophagy. Furthermore, p20BAP31 effectively decreased the expression level of p62 both in HCT116 and DLD-1 cells (Figure 1A–D). We further analyzed the expression of the Beclin-1 gene at mRNA levels and found that the expression levels of Beclin-1 were significantly higher after transfected with p20BAP31 (Figure 1E). Finally, we detected LC3B expression by immunofluorescence. The results exhibited that HCT116 and DLD-1 cells transfected with p20BAP31 both showed a dramatic and sustained increase in LC3B staining compared with a control (Figure 1F). These data demonstrate that p20BAP31 induces autophagy by promoting the convert of LC3I to LC3II and increasing the expression of Beclin-1.

### 2.2. The Effect of p20BAP31 on the PI3K-AKT-mTOR Pathway

**Figure 2:**
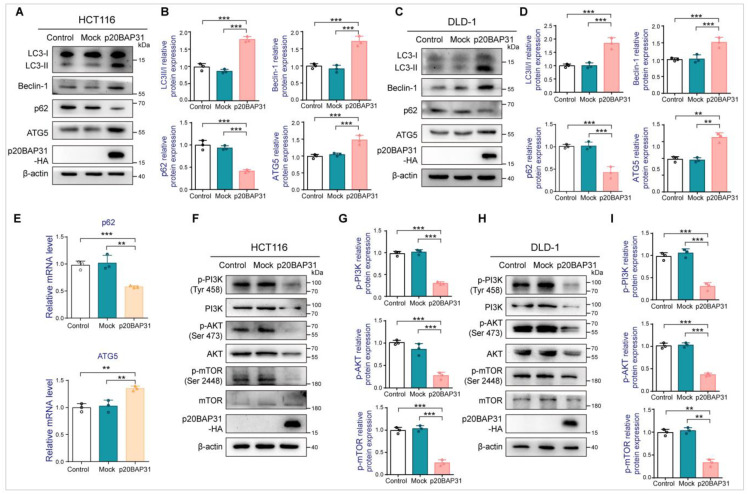
p20BAP31 induced autophagy by inhibiting the PI3K-AKT-mTOR pathway. (**A**,**B**) HCT116 cells were transfected with p20BAP31 for 48 h. The levels of LC3, Beclin-1, p62, ATG5 and p20BAP31-HA were analyzed by Western blot. β-actin was used as a loading control. (**C**,**D**) DLD-1 cells were transfected with p20BAP31 for 48 h. The levels of LC3, Beclin-1, p62, ATG5 and p20BAP31-HA were analyzed by Western blot. β-actin was used as a loading control. (**E**) The mRNA levels of p62 and ATG5 were detected by real-time PCR after transfected with p20BAP31 for 48 h in HCT116 cells. GAPDH served as a loading control. (**F**,**G**) The levels of p-PI3K, PI3K, p-AKT, AKT, p-mTOR, mTOR and p20BAP31-HA in HCT116 cells were analyzed by Western blot. β-actin was used as a loading control. (**H**,**I**) The levels of p-PI3K, PI3K, p-AKT, AKT, p-mTOR, mTOR and p20BAP31-HA in DLD-1 cells were analyzed by Western blot. β-actin was used as a loading control. The data are presented as means ± SDs, *n* = 3 replicates, by one-way ANOVA analysis. ** *p* < 0.01, *** *p* < 0.001.

To further explore the mechanism of p20BAP31 inducing autophagy in HCT116 and DLD-1 cells, we determined the expression of autophagy-related proteins. Consistent with the above results, p20BAP31 significantly enhanced the ratio of LC3II/LC3I and the expression level of Beclin-1 when compared with the control and mock groups both in HCT116 and DLD-1 cells (Figure 2A–D). Furthermore, p20BAP31 effectively decreased the autophagy substrate protein p62 and increased the expression of ATG5, which is involved in the conversion of LC3I to LC3II [24] (Figure 2A–D). We also analyzed the expression of p62 and ATG5 genes at mRNA levels. Indeed, p20BAP31 remarkably downregulated p62 and upregulated ATG5 at mRNA levels (Figure 2E). The PI3K-AKT-mTOR pathway is closely associated with autophagy; the inhibition of PI3K/Akt/mTOR pathway can stimulate autophagy [25]. To investigate the effect of p20BAP31 on the PI3K-AKT-mTOR signaling pathway, the phosphorylation levels of p-PI3K (Tyr 458), p-AKT (Ser 473) and p-mTOR (Ser2448) were determined in HCT116 and DLD-1 cells. As shown in Figure 2F–I, compared with the control and mock groups, p20BAP31 significantly decreased the phosphorylation levels of PI3K, AKT and mTOR. These results indicate that p20BAP31 induces autophagy through inhibiting the PI3K-AKT-mTOR pathway.

### 2.3. The Relationship between p20BAP31-Induced Autophagy and ER Stress

It has been reported that ER stress may initiate autophagy via the PERK pathway or the IRE1 pathway [39,40]. Since p20BAP31 stimulates the release of Ca^2+^ to trigger ER stress [12], we speculated that p20BAP31-induced ER stress might be a trigger for autophagy induction. To test this hypothesis, we applied a specific ER stress inhibitor 4-PBA, a molecular chaperone which reduces ER stress by improving protein folding [41]. The data showed that 4-PBA significantly inhibited p20BAP31-induced ER stress (Figure S2A,B). Additionally, 4-PBA downregulated the ratio of LC3-II/LC3-I and the expression of Beclin-1, and upregulated the expression of p62 (Figure S2C,D), which implies p20BAP31 induced autophagy by triggering ER stress.

**Figure 3:**
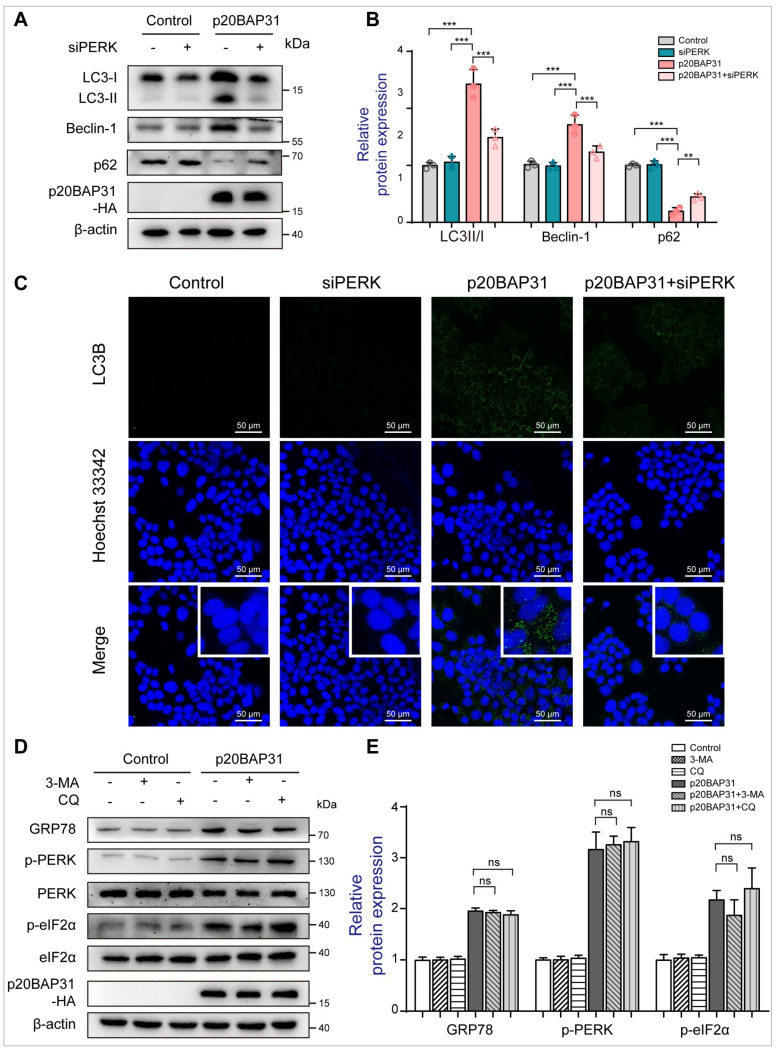
Inhibition of PERK attenuates p20BAP31-induced autophagy. (**A**) HCT116 cells were transfected with a PERK siRNA and p20BAP31 for 48 h. Western blot analysis was used to detect LC3, Beclin-1, p62 and p20BAP31-HA levels. (**B**) Quantification analysis of LC3, Beclin-1 and p62. β-actin served as the loading control. (**C**) HCT116 cells were transfected with a PERK siRNA and p20BAP31 for 48 h. Immunofluorescence staining of the autophagic marker LC3B (green). Hoechst 33342 (blue) was used to visualize cell nuclei. Representative images were captured using confocal immunofluorescence microscopy. (**D**,**E**) HCT116 cells were transfected with p20BAP31 for 48 h with or without pretreatment with 3-MA (50 μM) or CQ (15 μM) for 2 h. The levels of GRP78, p-PERK, PERK, p-eIF2α, eIF2α and p20BAP31-HA were analyzed by Western blot. The data are presented as means ± SDs, *n* = 3 replicates, by one-way ANOVA analysis. ** *p* < 0.01, *** *p* < 0.001, ns, not significance.

Given that the PERK pathway is closely associated with autophagy [42], and p20BAP31 activates UPR mainly through the PERK pathway, we used a specific siRNA to inactivate PERK and detect the effects of siPERK on p20BAP31-induced autophagy. We found that PERK knockdown effectively inhibited the PERK pathway, including p-PERK, p-eIF2α and ATF4 (Figure S3A,B). Consistent with 4-PBA treatment, PERK knockdown also remarkably inhibited the ratio of LC3-II/LC3-I and the expression of Beclin-1, and upregulated the expression of p62 (Figure 3A,B). Immunofluorescence staining assay further evidenced that siPERK decreased p20BAP31-induced expression of LC3B punctate-positive cells (Figure 3C). Moreover, inhibition of autophagy by 3-MA and CQ did not affect p20BAP31-induced ER stress (Figure 3D,E), further confirming that p20BAP31-induced ER stress is the upstream of autophagy. Collectively, the results show that p20BAP31 induces autophagy by triggering ER stress through the PERK pathway.

### 2.4. The Effect of ROS Accumulation on p20BAP31-Induced Autophagy

**Figure 4:**
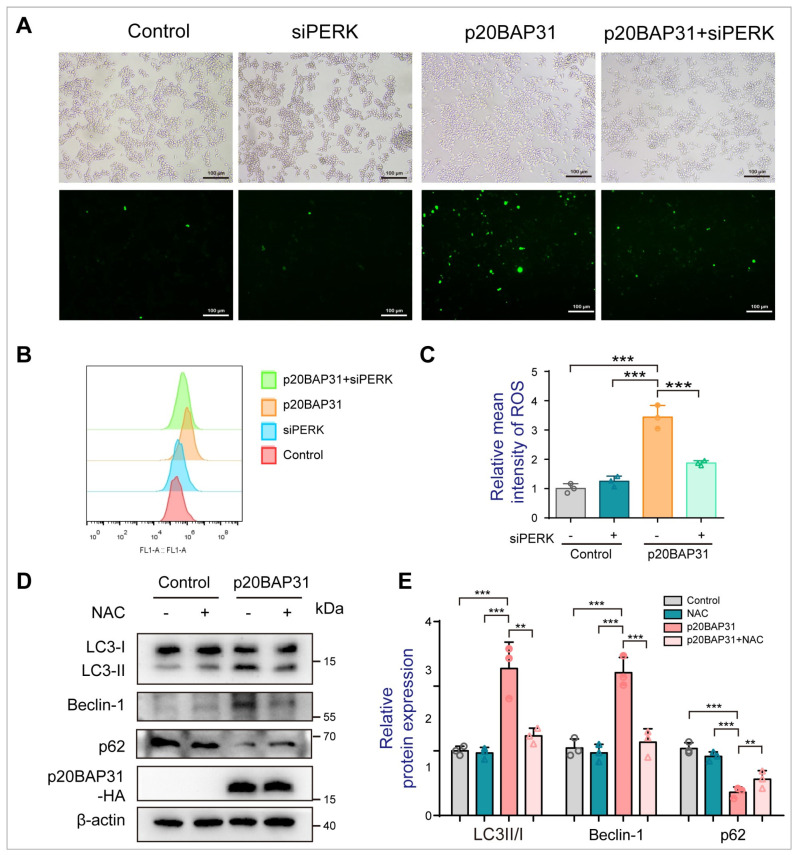
PERK-mediated ROS accumulation plays a vital role in p20BAP31-induced autophagy. (**A**) HCT116 cells were transfected with a PERK siRNA and p20BAP31 for 48 h. DCFH-DA fluorescence was observed by fluorescence microscopy. (**B**) The ROS content was detected by flow cytometry. (**C**) Quantitative analysis of ROS by flow cytometry. (**D**,**E**) HCT116 cells were transfected with p20BAP31 for 48 h with or without pretreatment with 5 mM NAC for 2 h. The levels of LC3, Beclin-1, p62 and p20BAP31-HA were analyzed by Western blot. The data are presented as the means ± SDs of three independent experiments. ** *p* < 0.01 and *** *p* < 0.001.

Considering the activation of the PERK signaling pathway contributes to ROS induction [43], and p20BAP31 promotes the accumulation of ROS [14], we detected the effects of PERK on p20BAP31-induced ROS production. The fluorescence intensity and the flow cytometry assay both showed that PERK knockout effectively reduced the accumulation of intracellular ROS induced by p20BAP31 (Figure 4A–C). Importantly, Western blot results showed that NAC (a ROS scavenger) suppressed p20BAP31-induced autophagy, by decreasing the ratio of LC3-II/LC3-I and the expression of Beclin-1, and increasing the expression of p62 (Figure 4D,E). These data illustrate that p20BAP31 induces autophagy via PERK-mediated ROS accumulation.

### 2.5. Analysis of Autophagy Inhibition Induced by p20BAP31 on Cell Apoptosis

**Figure 5:**
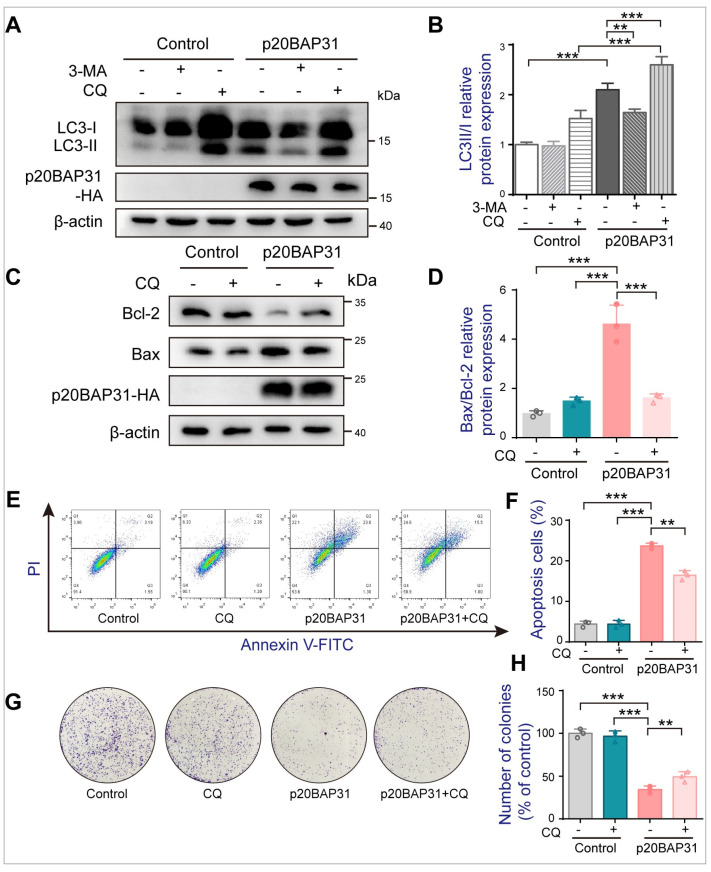
Inhibition of autophagy attenuates p20BAP31-induced apoptosis. HCT116 cells were transfected with p20BAP31 for 48 h with or without pretreatment with 3-MA (50 μM) or CQ (15 μM) for 2 h. (**A**) Western blot analysis was used to detect LC3 and p20BAP31-HA levels. (**B**) Quantification analysis of LC3II/LC3I. β-actin served as a loading control. (**C**,**D**) The levels of Bcl-2, Bax and p20BAP31-HA were analyzed by Western blot. β-actin was used as a loading control. (**E**) Apoptosis was determined by flow cytometry analyses of Annexin V-FITC/PI double-staining. (**F**) Quantitative analysis of apoptotic rate by flow cytometry. (**G**) The colony formation assay was used to determine the colony formation ability of HCT116 cells with or without CQ under the treatment of p20BAP31. (**H**) The quantitation graph indicates the colony formation of HCT116 cells. The data are presented as means ± SDs, *n* = 3 replicates, by one-way ANOVA analysis. ** *p* < 0.01, ***p < 0.001.

The relationship between autophagy and apoptosis is complicated. Considering that our recent studies have confirmed that p20BAP31 induces apoptosis, while in this study, p20BAP31 induces autophagy, it is necessary to explore the relationship between p20BAP31-induced apoptosis and autophagy. Accordingly, we determined the effect of autophagy inhibitors, such as 3-methyladenine (3-MA) and chloroquine (CQ) on p20BAP31-mediated autophagy in HCT116 cells. As shown in Figure 5A,B, pretreatment with 3-MA resulted in the decrease of LC3-II/LC3-I compared with the p20BAP31 group in HCT116 cells. Meanwhile, the inhibition of autophagy by CQ, an autophagy inhibitor that prevents autolysosomal degradation [44], enhanced the expression of LC3-II/LC3-I compared with p20BAP31 use alone. These results indicated that both 3-MA and CQ inhibited p20BAP31-induced autophagy. Importantly, CQ partially suppressed p20BAP31-induced elevated ratio of Bax/Bcl-2 (Figure 5C,D) and p20BAP31-induced apoptosis in HCT116 cells (Figure 5E,F), suggesting that inhibition of autophagy attenuates p20BAP31-induced apoptosis. Moreover, the colony-forming assay also showed that CQ alleviated p20BAP31-induced inhibition of cell proliferation in HCT116 cells (Figure 5G,H). These results indicate that p20BAP31 relieves the inhibitory effect of p20BAP31 on HCT116 cells by inhibiting autophagy.

### 2.6. Overexpression of p20BAP31 in CRC Induces Autophagy and Inhibits Tumor Growth In Vivo

**Figure 6:**
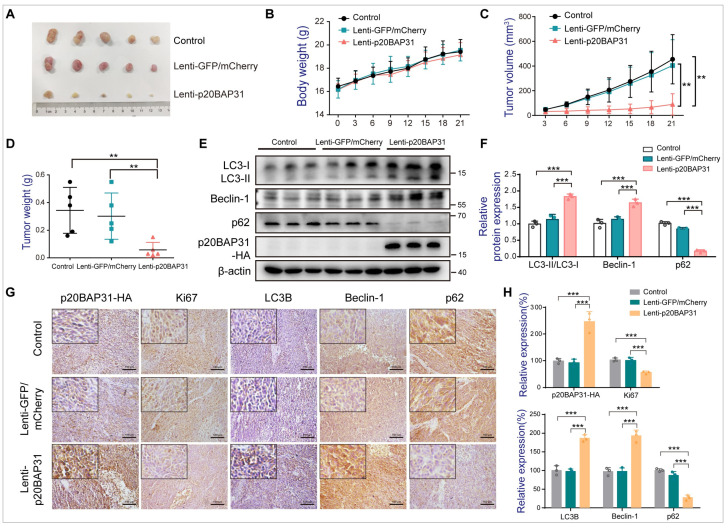
Overexpression of p20BAP31 in CRC induces autophagy and inhibits tumor growth in vivo. (**A**) The mice were randomly divided into three groups: control, lenti-GFP/mCherry and lenti-p20BAP31. Representative image of tumors at day 21. (**B**) Body weight of the three groups. (**C**) Tumor volume was measured in the three groups once every three days and plotted. (**D**) Tumor weights of the three groups at the end of experiment. (**E**) Western blot analysis was used to detect LC3, Beclin-1, p62 and p20BAP31-HA levels in the HCT116 xenografts following the indicated treatment. (**F**) Quantification analysis of LC3, Beclin-1 and p62. β-actin served as the loading control. (**G**) Immunohistochemistry staining for p20BAP31-HA, Ki67, LC3B, Beclin-1 and p62 in the tumor specimens from the mice. Representative images were provided as indicated. (**H**) Relative expression of p20BAP31-HA, Ki67, LC3B, Beclin-1 and p62 in tumor tissues of the three groups. The values are presented as means ± SDs, *n* = 5 mice per group, by one-way ANOVA analysis. ** *p* < 0.01, *** *p* < 0.001.

To further assess the function of p20BAP31 in autophagy-induced cell death, the effect of p20BAP31 was observed in nude mice bearing HCT116 xenograft. p20BAP31 overexpression did not affect the body weight of the mice, but significantly inhibited tumor growth compared with the control and lenti-GFP/mCherry groups (Figure 6A–D). Consistently, stable p20BAP31 overexpression enhanced the ratio of LC3-II/LC3-I and the expression of Beclin-1, and decreased the expression of p62, as compared with the control and lenti-GFP/mCherry groups (Figure 6E,F). In addition, Ki67 staining of tumor sections was used to detect the proliferation in vivo; the results demonstrated that p20BAP31 reduced Ki67 expression levels in tumor tissues (Figure 6G,H). Consistent with the results observed in vitro, p20BAP31 significantly upregulated the ratio of LC3-II/LC3-I and the expression of Beclin-1, and decreased the expression of p62 (Figure 6G,H). Collectively, these results indicate that p20BAP31 inhibits tumor growth through inducing autophagic cell death.

## 3. Discussion

Previous studies have shown that p20BAP31 induces apoptosis through multiple pathways, suggesting the potential value of p20BAP31 in antitumor therapy research [14]. However, whether p20BAP31 induces autophagic cell death is still unknown; the relationships among p20BAP31-induced autophagy, apoptosis and ER stress are poorly understood. In this study, we demonstrate that p20BAP31 induces autophagy through inhibition of the PI3K/AKT/mTOR pathway and activation of PERK-mediated ROS accumulation, which promotes apoptosis and eventually leads to cell death.

Autophagy is a highly conserved catabolic process involved in a variety of cell biological activities [45]. Although previous study has reported that the loss of BAP31 induces autophagy [11], whether p20BAP31 induces autophagy still unknown. In this study, p20BAP31 significantly increased the expressions of autophagy-related proteins Beclin-1 and ATG5, and promoted the turnover of LC3-II and p62 degradation in HCT116 and DLD-1 cells. In addition, the PI3K/AKT/mTOR signaling pathway is widely acknowledged as a major pathway involved in the initiation and regulation of autophagy [46,47]. Our results indicated that p20BAP31 dramatically decreased the levels of phosphorylated PI3K, AKT and mTOR. These results suggest that p20BAP31 induces autophagy via suppressing the PI3K/Akt/mTOR signaling pathway in colorectal cells. Furthermore, it has been reported that the protein kins-3-phosphoinositide-dependent kinase 1 (PDPK1) is essential for the activation of the PI3K/AKT/mTOR pathway. PDPK1 interacts with AKT and phosphorylates it at Thr-308 dependent on the PH domain [48]. Therefore, we speculate whether p20BAP31 regulates the PI3K/AKT/mTOR pathway through regulating PDPK1. However, the molecular mechanism by which p20BAP31 inhibits the PI3K/AKT/mTOR pathway remains to be further explored.

Numerous studies have demonstrated that ER stress triggers autophagy [33,34]. p20BAP31 has shown to induce ER stress by stimulating the release of ER Ca^2+^ [12]. Therefore, we next explore the relationship between p20BAP31-induced ER stress and autophagy. The results showed that the ER stress inhibitor 4-PBA effectively alleviated p20BAP31-induced autophagy by inhibiting the ER stress response, suggesting that ER stress is at least partially involved in p20BAP31-induced autophagy. In addition, the PERK–eIF2α pathway is critical for autophagy induction after ER stress, further confirming the link between autophagy and UPR [49]. Considering that p20BAP31 induced ER stress mainly by activating the PERK pathway, we further investigate the effects of PERK on p20BAP31-induced autophagy. We used a specific siRNA to inactivate PERK, and found that siPERK remarkably inhibited p20BAP31-induced autophagy, implying that PERK is required for p20BAP31-induced autophagy. Furthermore, the accumulation of ROS plays an important role in apoptosis and autophagy [50,51]. We have demonstrated that p20BAP31 induced apoptosis by promoting PERK-mediated ROS production, and our results further proved that NAC (an ROS scavenger) significantly reduced p20BAP31-induced autophagy. Collectively, these findings suggest that PERK-mediated ROS accumulation plays a crucial role in p20BAP31-induced autophagy.

To further study the relationship between p20BAP31-induced autophagy and ER stress, we used autophagy inhibitors 3-MA and CQ. Our results proved that the inhibition of early autophagosome formation with 3-MA or inhibition of late autolysosome formation with CQ both prevented p20BAP31-induced autophagy. Nonetheless, the hindrance of autophagy through 3-MA and CQ did not affect p20BAP31-induced ER stress. Combined with the above results, inhibiting ER stress effectively reduces p20BAP31-induced autophagy, further identifying ER stress activation as an upstream event that induces autophagy in HCT116 cells transfected with p20BAP31. Moreover, the functional relationship between apoptosis and autophagy is complicated; apoptosis and autophagy may activate or inhibit each other [31,32]. We have demonstrated that p20BAP31 induces both apoptosis and autophagy; thus, it is necessary to study the relationship between p20BAP31-induced autophagy and apoptosis. Our results indicated that the autophagy inhibitor CQ significantly inhibited p20BAP31-induced apoptosis and enhanced the inhibitory effect of p20BAP31 on the growth of HCT116 cells. Taken together, these results reveal the relationship between ER stress, autophagy and apoptosis induced by p20BAP31. p20BAP31-induced ER stress may be the upstream of autophagy, and then promotes p20BAP31-induced apoptosis, eventually leading to cell death.

In addition, to support and validate the results in vitro with more reliable evidence, we tested the effect of p20BAP31 on a xenograft nude mouse model. We found that p20BAP31 showed effectively inhibitory effects on tumor growth. Western blot and immunohistochemistry analysis confirmed p20BAP31 significantly upregulated the expression of LC3B and Beclin-1, and downregulated the expression of p62. These results demonstrated that p20BAP31 inhibits tumor growth through inducing autophagic cell death.

**Figure 7:**
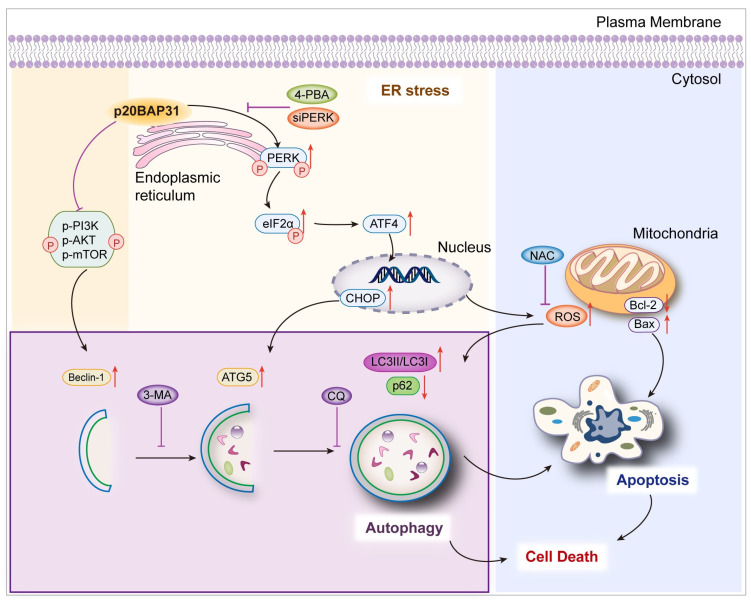
Schematic model of the proposed mechanisms of p20BAP31 in modulating autophagy, ER stress and apoptosis. See text for details.

In summary, the research on p20BAP31 is still being deeply explored, and its potential application to clinical diagnosis is of great significance. Furthermore, the property of p20BAP31 to induce cell death through multiple pathways gives it a great advantage in anti-tumor. A comprehensive investigation of the function of p20BAP31 is helpful for the clinical diagnosis and treatment of colorectal cancer, and provides a theoretical foundation for the development of novel therapeutic approaches. In this study, the data presented herein suggest the relationships among p20BAP31-induced autophagy, ER stress and apoptosis in colorectal cells. A schematic drawing of the proposed mechanisms is shown in Figure 7. We propose that (Ⅰ) p20BAP31 induces autophagy via suppressing the PI3K/AKT/mTOR signaling pathway in HCT116 and DLD-1 cells; (Ⅱ) inhibition of ER stress through the PERK pathway reduces p20BAP31-induced autophagy; (Ⅲ) PERK-mediated ROS accumulation is essential for p20BAP31-induced autophagy; and (Ⅳ) p20BAP31-induced autophagy promotes apoptosis and ultimately leads to cell death. These results provide a more comprehensive insight into the molecular mechanisms of p20BAP31-induced cell death.

## 4. Materials and Methods

### 4.1. Reagents and Antibodies

ER stress inhibitor 4-phenylbutyric acid (4-PBA, cat. no. 1821-12-1, 99.98%), autophagy inhibitors 3-Methyladenine (3-MA, cat. no. 5142-23-4, 99.91%) and Chloroquine (CQ, cat. no. 54-05-7, 99.82%) were purchased from Med Chem Express (Shanghai, China). ROS scavenger N-Acetyl-L-cysteine (NAC, cat. no. 616-91-1, 99%) was purchased from Beyotime (Shanghai, China). Primary antibodies against LC3B, GRP78, HA, β-actin were purchased from Proteintech (Wuhan, China). Primary antibody against Beclin-1 was purchased from Affinity (Changzhou, China). Primary antibodies against p62, ATG5, Ki67 and ATF4 were purchased from Beyotime. Primary antibodies against Bcl-2 and Bax were purchased from Wanleibio (Shenyang, China). Primary antibodies against PI3K, phospho-PI3K, AKT, phospho-AKT, mTOR, phospho-mTOR, PERK, eIF2α and phospho-eIF2α were purchased from Cell Signaling Technology (Dancers, MA, USA). Primary antibody against phospho-PERK was purchased from SAB (Greenbelt, MD, USA). The secondary antibodies, HRP-linked anti-mouse IgG, HRP-linked anti-rabbit IgG were purchased from Cell Signaling Technology. The secondary antibody, FITC goat anti-rabbit IgG (H + L) antibody was purchased from Apexbio (Shanghai, China). The adenovirus expressing mCherry-GFP-LC3B fusion protein (Ad-mCherry-GFP-LC3B) was purchased from Beyotime.

### 4.2. Cell Culture

The human colorectal carcinoma cell lines HCT116 (cat. no. TCHu99) and DLD-1 (cat. no. TCHu134) were purchased from the cell bank of the Chinese Academy of Sciences (Shanghai, China). The cell lines were cultured in DMEM (BasalMedia, Shanghai, China) supplemented with 10% fetal bovine serum (FBS), 1% L-glutamine and 1% penicillin-streptomycin (Gibco) in a 5% CO_2_ incubator (Thermo Fisher Scientific, Waltham, MA, USA) at 37 °C. All cell lines were confirmed to be mycoplasma-free by Hoechst DNA staining (indirect method).

### 4.3. Plasmid Construction and Transfection

The pcDNA3.1(-) plasmid (Mock) was obtained from Thermo Fisher Scientific. Plasmid encoding p20BAP31 (aa 1–164 of human BAP31) with a COOH-terminal HA tag was cloned into the EcoRI and BamHI sites of the vector pcDNA3.1 (-) (PCR primer sequences are shown in Table S1). Cells were transfected using Lipo8000™ transfection reagent (Beyotime). Briefly, the plasmid(μg): Lipofectamine (μL) ratio was 1:1.5, the plasmids were mixed in Opti-MEM and then Lipo8000™ transfection reagent was added. The solution was mixed gently, and then added to the plate dropwise. After 6 h, the medium was replaced and the cells harvested at appropriate times.

### 4.4. Small Interfering RNA (siRNA) Transfection

HCT116 cells were transfected with siRNA for PERK (GenePharma, Suzhou, China) using Lipo8000™ transfection reagent (Beyotime). Briefly, the siRNA (pmol): Lipofectamine (μL) ratio was 35:1, the plasmids were mixed in Opti-MEM, Lipo8000™ transfection reagent was added and mixed gently and then was added to the plate dropwise. After 6 h, it was replaced with the fresh medium and the cells were harvested at appropriate times. The siRNAs sequences are shown in Table S1.

### 4.5. Western Blot Analysis

The cells and tissues were lysed in RIPA buffer (Beyotime) containing 1% protease inhibitor cocktail and 1 mM PMSF (Sigma-Aldrich, St. Louis, MO, USA) for 30 min. Protein concentration was determined using a BCA Protein Assay Kit (Beyotime). Equal amounts of protein were separated by 10–15% SDS-PAGE, and then transferred to polyvinylidene difluoride (PVDF) membranes (Merk Millipore, Darmstadt, Germany). After being blocked in 5% fat-free milk, the PVDF membrane was incubated with the corresponding primary antibodies at 4 °C overnight, then incubated with HRP conjugated secondary antibodies. The protein bands were visualized with a Bio-Rad ChemiDoc ^TM^ imaging system with an ECL detection kit (Thermo Fisher Scientific). The Image Lab software (5.2.1, Bio-Rad, Hercules, CA, USA) was utilized for quantification.

### 4.6. Real-Time PCR Analysis

Total RNA was extracted from HCT116 cells with Trizol reagents (Sigma-Aldrich, St. Louis, MO, USA). One microgram of total RNA was used for reverse transcription into cDNA (Promega, Madison, USA). Quantitative real-time PCR (qRT-PCR) was carried out using 2× SYBR Green qPCR Master Mix (Cwbio, Taizhou, China). The relative expression of mRNA was evaluated with the 2^−ΔΔCt^ method, and normalized to GAPDH. The qRT-PCR primers for LC3B, Beclin-1, p62, ATG5 and GAPDH are listed in Table S1.

### 4.7. Immunofluorescence Assay

The cells were fixed in 4% paraformaldehyde (PFA), permeabilized with 0.2% Triton X-100, blocked with 1% bovine serum albumin (BSA) at room temperature, and then incubated with anti-LC3B primary antibody (1:100 dilution) at 4 °C overnight. The FITC goat anti-rabbit IgG (H + L) secondary antibody (Apexbio) was incubated at room temperature for 2 h in the dark. The nuclei were stained with Hoechst 33342 for 5 min. An inverted confocal microscopy (DM6000CS, Leica, Wetzlar, Germany) was used to capture the images.

### 4.8. Cell Apoptosis Assay

Cell apoptosis was detected by flow cytometric analysis using the Annexin V-FITC Apoptosis Detection Kit (Beyotime). Briefly, after transfection or treatment, the cells were harvested and washed repeatedly with cold PBS, and then resuspended in 100 μL 1× binding buffer. Cells were incubated with 4 μL Annexin V-FITC reagent and 8 μL PI for 15 min in the dark at room temperature. Finally, 400 μL 1× binding buffer was added to the stained cells and analyzed using a FACScan flow cytometer (BD Biosciences, San Jose, CA, USA).

### 4.9. Colony Forming Assay

Cells were seeded into 6-well plates (2000 cells per well). After pretreatment with or without CQ and transfected with or without p20BAP31, cells were cultivated for 10 days, then fixed with 4% formaldehyde for 30 min and stained with 0.1% crystal violet for 15 min. Finally, the plates were washed with running water and air-dried at RT. All samples were performed in triplicate. Colony count analysis was performed with ImageJ software (v. 1.8.0) (NIH, Bethesda, MD, USA).

### 4.10. Measurement of Intracellular ROS Levels

ROS levels were detected by the Reactive Oxygen Species Assay Kit (Beyotime) according to the protocol. After transfection with p20BAP31 or siPERK, the cells were stained with DCFH-DA (10 μM) at 37 °C in the darkness for 20 min, then washed with serum-free DMEM three times. The cells were collected for ROS analysis by a FACScan flow cytometer (BD Biosciences). Furthermore, ROS levels were also determined by fluorescence microscopy. After the appropriate treatment, the cells were stained with DCFH-DA (10 μM) and then washed in accordance with the above methods. The images were photographed with a DMI3000B fluorescence microscope (Leica, Wetzlar, Germany).

### 4.11. Tumor Xenografts

We chose 5-week-old male BALB/c nude mice purchased from Changsheng Biotechnology Co., Ltd. (Benxi, China) for tumor xenografts experiments. All mice were raised in specific pathogen-free conditions, free access to water and food, and a 12 h light/dark schedule at 25 ± 1 °C. The mice were randomly divided into three groups; each group included five mice (n = 5). HCT116 cells were transfected with lentivirus-EGFP/mCherry or lentivirus-p20BAP31 (VectorBuilder, Guangzhou, China), and selected by puromycin (5 μg/mL) for 2–3 weeks. HCT116 cells (5 × 10^6^ cells/100 μL) stably expressing p20BAP31 (Lenti-p20BAP31) or the control vector (Lenti-EGFP/mCherry) were injected subcutaneously into the nude mice. Tumor volumes and body weights were monitored every 3 days. Tumor volume was determined by a caliper and calculated by formula: V (mm^3^) = (length × width^2^)/2. All mice were sacrificed after injection of HCT116 cells for 21 days. Tumor tissue was harvested and weighed.

### 4.12. Immunohistochemistry Analysis

Paraffin-embedded tumor tissues sections of 5 μm thickness were dewaxed in xylene and rehydrated by a graded series of ethanol. After antigen retrieval and inactivated with H_2_O_2_ (3%), the sections were incubated in 5% BSA for 20 min and incubated with primary antibody at 4 °C overnight. The next day, sections were washed extensively and incubated with a biotinylated secondary antibody at 37 °C for 1 h. Finally, the sections were visualized with diaminobenzidine (DAB), stained with hematoxylin, dried and cover-slipped with neutral balsam. The sections were photographed under bright field using a DMI3000B fluorescence microscope.

### 4.13. Statistical Analysis

The difference between two groups was conducted using an unpaired two-tailed Student’s *t*-test, and statistical analyses among groups were performed by one-way analysis of variance (ANOVA) and least significant difference (LSD) post hoc test by SPSS v.17.0 (IBM, NY, USA). The data were repeated at least three times and presented as the mean ± standard deviation (SD). GraphPad Prism (Prism 7.0, San Diego, CA, USA) was used for all histograms. *p* values < 0.05 were considered statistically significant.

## 5. Conclusions

This study demonstrated that p20BAP31 induces autophagy in colorectal cells and further explored the potential molecular mechanisms. p20BAP31 induces autophagy by inhibition of the PI3K/AKT/mTOR signaling and activation by PERK-mediated ROS production, and further promoting p20BAP31-induced apoptosis. Furthermore, p20BAP31 inhibited tumor growth in vivo through inducing autophagy. In light of this, exploring the relationship among ER stress, autophagy and apoptosis induced by p20BAP31 also provides a possibility for its application in cancer therapy.

## Data Availability

The data used to support the findings of this study are available from the corresponding authors upon request.

## Supplementary Materials

The following supporting information can be downloaded at: https://www.mdpi.com/article/10.3390/ijms25105101/s1.
