# Supplementary material for: p20BAP31 Induces Autophagy in Colorectal Cancer Cells by Promoting PERK-Mediated ER Stress
Source: Int J Mol Sci. 2024 May 7;25(10):5101. doi: 10.3390/ijms25105101 (PMC11121724; doi:10.3390/ijms25105101)
Supplement: Supplementary file 1 [file ijms-25-05101-s001.zip › ijms-2990808-supplementary.pdf]

p20BAP31 Induces Autophagy in Colorectal Cancer Cells by Promoting PERK-Mediated ER Stress

Xiaohan Jiang, Guoxun Li, Benzhi Zhu, Jiaying Yang, Shuyu Cui, Rui Jiang * and Bing Wang *

College of Life and Health Science, Northeastern University, 195 Chuangxin Road, Hunnan District, 110819 Shenyang, China; xiaohanjiang2014@163.com (X.J.); liguoxuner@126.com (G.L.); zhubenzhi0203@163.com (B.Z.); 2201476@stu.neu.edu.cn (J.Y.); cuishuyu0615@163.com (S.C.)

* Correspondence: jiangrui@mail.neu.edu.cn (R.J.); wangbing@mail.neu.edu.cn (B.W.)

Supplementary Information

Summary

Figure S1–S3 and the corresponding figure legends.

Table S1: Sequences of primers for PCR, qRT-PCR and siRNA.

Supplementary Figures and Figure legends

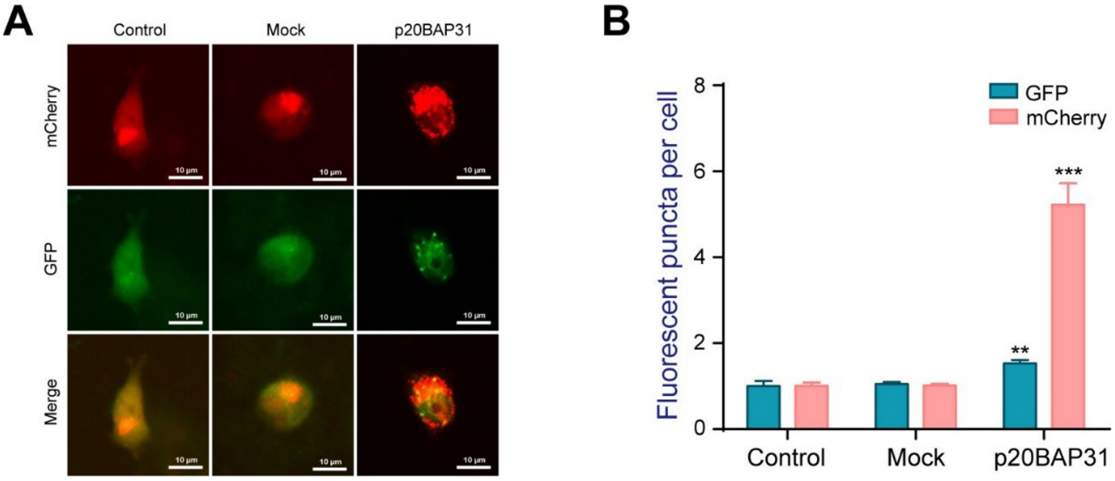

Figure S1. Overexpression of p20BAP31 induces autophagy. (**A**) HCT116 cells were transfected with p20BAP31 and Ad-mCherry-GFP-LC3B for 48 h. Representative images were captured by a fluorescence microscope. (**B)** Quantification analysis of EGFP and mCherry fluorescent puncta (n = 10 cells. $**P < 0.01$, $***P < 0.001$.

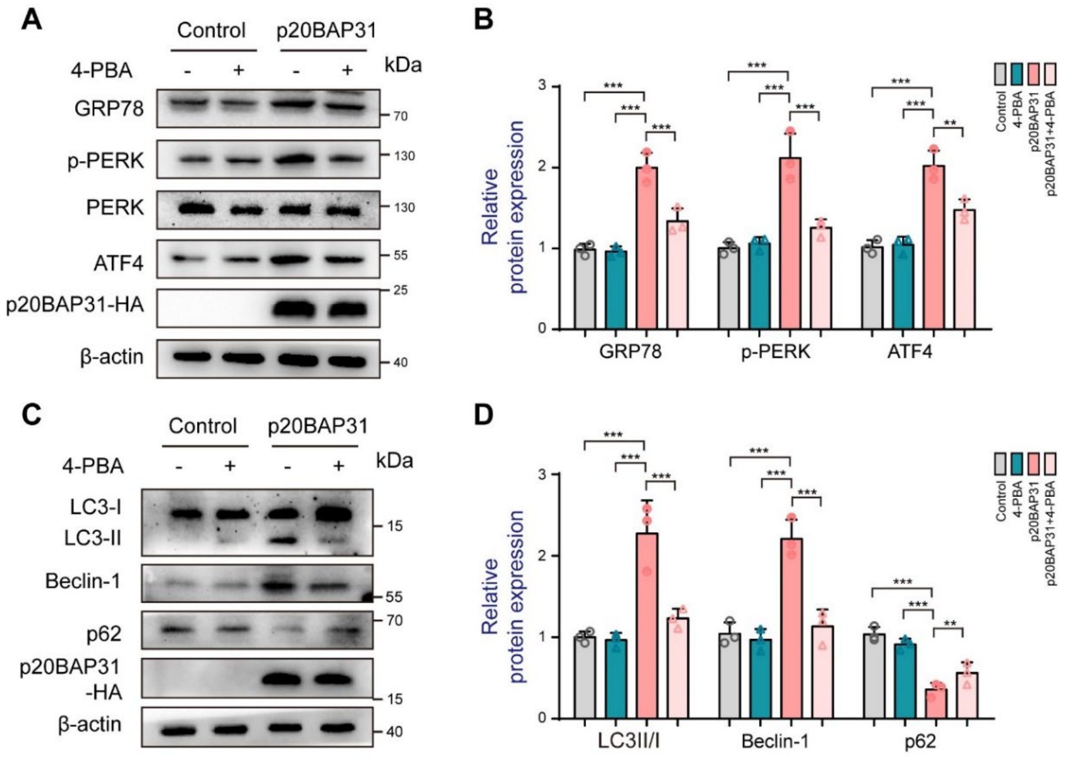

Figure S2. Inhibition of ER stress attenuates p20BAP31-induced autophagy. HCT116 cells were transfected with p20BAP31 for 24 h with or without pretreatment with 200 μM 4-PBA for 2 h. (**A**) Western blot analysis was used to detect GRP78, p-PERK, PERK, ATF4 and p20BAP31-HA levels. (**B**) Quantification analysis of GRP78, p-PERK and ATF4. β-actin served as the loading control. (**C**) Western blot analysis was used to detect LC3, Beclin-1, p62 and p20BAP31-HA levels. (**D**) Quantification analysis of LC3, Beclin-1 and p62. β-actin served as the loading control. The data are presented as the means ± SDs of three independent experiments. $^{**}P < 0.01$, $^{***}P < 0.001$, ns, not significance.

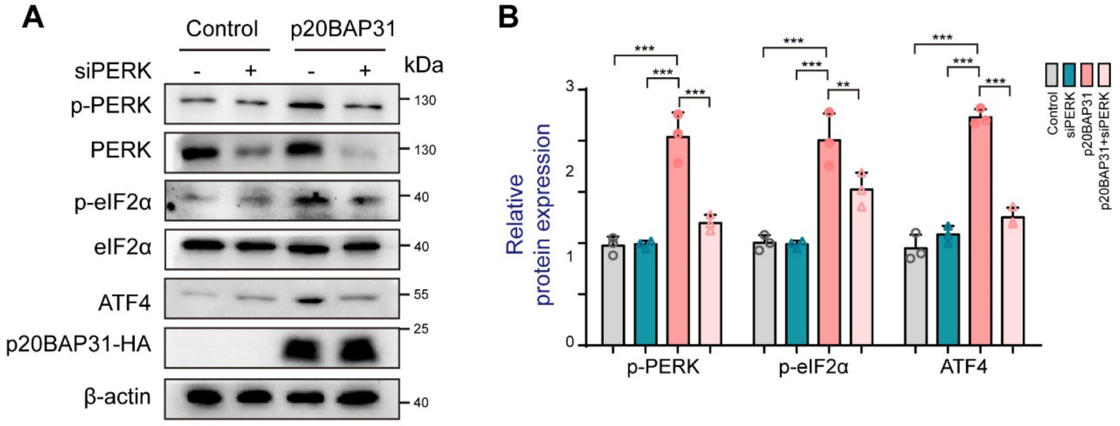

Figure S3. siPERK inhibits the activation of PERK signaling pathway induced by p20BAP31. HCT116

cells were transfected with a PERK siRNA and p20BAP31 for 48 h. (**A**) Western blot analysis was used to detect p-PERK, PERK, p-eIF2α, eIF2α, ATF4 and p20BAP31-HA levels. (**B**) Quantification analysis of p-PERK, p-eIF2α and ATF4. β-actin served as the loading control. The data are presented as the means ± SDs of three independent experiments. $**P < 0.01$, $***P < 0.001$.

Table S1. Sequences of primers for PCR, qRT-PCR and siRNA.

Targets	Primer Sequences (5'-3')	Purpose
Human p20BAP31 Forward	CGGAATTCATGAGTCTGCAGTGGACTGC	PCR
Human p20BAP31 Reverse	CGGGATCCTTAAGCGTAGTCTGGGACGTCGTATGGGT AGTCAACAGCAGCTCCCTTCT	PCR
Human LC3B Forward	ACACAGCATGGTCAGCGTCT	qRT-PCR
Human LC3B Reverse	TTTCATCCCGAACGTCTCCT	qRT-PCR
Human Beclin-1 Forward	AACCTCAGCCGAAGACTGAA	qRT-PCR
Human Beclin-1 Reverse	GACGTTGAGCTGAGTGTCCA	qRT-PCR
Human p62 Forward	AGCGTCTGCGAGGGAAAG	qRT-PCR
Human p62 Reverse	ACCCGAAGTGTCCGTGTTT	qRT-PCR
Human ATG5 Forward	AAGACCTTCTGCACTGTCCA	qRT-PCR
Human ATG5 Reverse	GAGTTTCCGATTGATGGCCC	qRT-PCR
Human GAPDH Forward	TCGTGGAAGGACTCATGACC	qRT-PCR
Human GAPDH Reverse	ATGATGTTCTGGAGAGCCCC	qRT-PCR
Human siPERK sense	GGUUGGAGACUUUGGGUUAUU	siRNA
Human siPERK antisense	UAACCCAAAGUCUCCAACCUU	siRNA
